# Successful preoperative diagnosis of heterotopic pancreas in the duodenum

**DOI:** 10.1016/j.ijscr.2019.01.026

**Published:** 2019-01-30

**Authors:** Ken Min Chin, Damien M.Y. Tan, Norman H.L. Chan, Brian K.P. Goh

**Affiliations:** a Department of Hepatopancreatobiliary and Transplant Surgery, Singapore General Hospital, Singapore; b Department of Gastroenterology and Hepatology, Singapore General Hospital, Singapore; c Department of Pathology, Singapore General Hospital, Singapore; d Duke-National University of Singapore (NUS) Medical School, Singapore

**Keywords:** Heterotopic pancreas, Intra-abdominal, Malignant

## Abstract

**Introduction:**

Heterotopic pancreas (HP) is a relatively rare entity occurring in approximately 5% of the general population. It most commonly presents as an asymptomatic mass incidentally picked up on unrelated scans. HP most commonly occurs intra-abdominally, but has been known to occur in extra-abdominal sites such as the lung and brain. It is widely considered to bear little to no malignant potential. Difficulty and ambiguity in the diagnosis of HP commonly results in interventional dilemma and delay.

**Presentation of case:**

We present a case of uncomplicated HP that was ultimately treated conservatively.

**Discussion:**

A literature review is made of the typical workup in a patient with suspected HP, and the characteristic radiological and endoscopic findings commonly used for diagnosis of this rare condition. A succinct summary of management guidelines for HP is reviewed.

**Conclusion:**

HP is most commonly an incidental finding. Ambiguity surrounding its diagnosis commonly gives rise to interventional dilemma and delay. The gold standard for diagnosis remains that of EUS and FNA with histological confirmation. This report has been written in concordance with the SCARE criteria Agha et al. [1].

## Highlights

• HP mostly occurs in the alimentary tract, with a predilection for the stomach and duodenum.
• Radiological features of HP include endoluminal growth pattern, ill-defined borders, enhancement of overlying mucosa.
• Endoscopic features of HP include lobulated margins, anechoeic ductal structures and central umbilication.
• Management of HP is controversial and commonly delayed due to diagnostic dilemma.

## 1 Introduction

Heterotopic pancreas (HP) is defined as pancreatic tissues lacking vascular or anatomic communication with anatomical pancreas, but possessing histological features of acinic and islet cell formation, ductal development, and independent blood supply. It is present in approximately 5% of the general population.

It is classified into 4 subtypes based on Gasper-Fuentes’ modification of Heinrich’s initial classification [2]: Type I consists of typical pancreatic tissue. Type II consists of pancreatic ducts only. Type III consists of acinic cells only. Type IV consists of islet cells only. This report highlights a typical sequence of investigations in the workup and diagnosis of an asymptomatic patient with HP picked up incidentally.

## 2 Case report

This case involves a 66-year-old gentleman with hypertension. He presented with elevated Cancer Antigen 19-9 levels of 128 μ/m incidentally detected on routine screening. Liver function test and other tumor markers (carcinoembryonic antigen, alpha-fetoprotein, cancer antigen 125) were within normal limits.

**Fig. 1:**
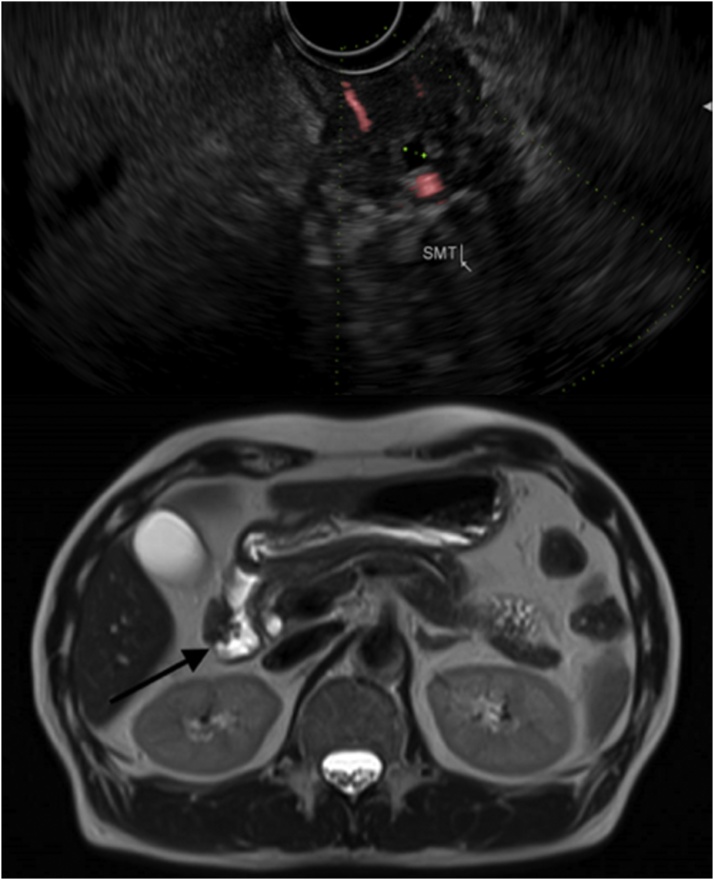
Green markers represent a 0.19 cm wide, anechoic central duct-like structure seen within the 1.8 × 0.9 cm submucosal mass on EUS (top). Black arrow demarcates the 1.7 × 2.0 cm lobulated, endoluminal soft tissue mass abutting the lateral wall of the junction between the first (D1) and second (D2) part of the duodenum as seen on MRCP (bottom).

**Fig. 2:**
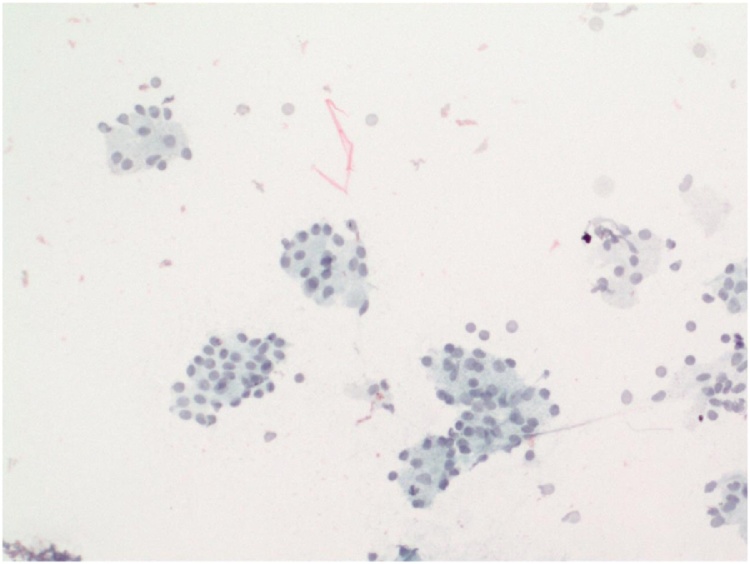
Demonstration of streaks of acinar cells of pancreatic morphology, ducts with intervening connective tissue seen on pathological analysis of fine needle aspirate tissues stained with Hematoxylin and Eosin (H&E).

Magnetic resonance cholangiopancreatogram (MRCP) revealed a lobulated, ill-defined endoluminal soft tissue mass measuring 1.7 × 2.0 cm abutting the lateral wall of the junction between the first (D1) and second (D2) part of the duodenum, but not invading into mucosa. Anatomical pancreatic tissue had no ductal dilatation, and was similar in appearance and consistency to the identified mass (Fig. 1). Differentials at this point included heterotopic pancreas, and other mucosal/submucosal malignancies such as gastrointestinal stromal tumor (GIST). He was further investigated with endoscopic ultrasound (EUS). The D1/D2 junction intramural (submucosal) lesion was identified and measured 1.8 cm × 0.9 cm. It had lobulated margins, acinar cells and an anechoic 0.2 cm central duct-like structure, all suggestive of heterotopic pancreas (Fig. 1). Fine needle aspiration (FNA) revealed streaks of acinar cells of pancreatic morphology and ducts with intervening connective tissue, diagnostic of heterotopic pancreas (Fig. 2).

Given that this patient was asymptomatic with no malignant features seen on investigations, he was managed conservatively. He has since been followed-up in clinic once at an 8-month interval with a repeat CTAP showing a stable mass and no enlargement or invasion.

## 3 Discussion

According to literature, HP is a rare occurrence found in 0.5–14% of all autopsies performed and 0.5% of all upper abdominal laparotomies [3,4]. The first reported case in history was documented in 1729 by Schultz, but the first histological confirmation was not until 1859 by Klob [4,5]. HP has a predilection for the stomach (25–30%), duodenum (15–30%) and jejunum (15–20%), but can be found anywhere along the gastrointestinal tract. Extra-intestinal occurrences of HP include the liver, biliary tract, ampulla of Vater, gallbladder, Meckel’s diverticulum, umbilicus, fallopian tubes, pelvis, mesocolon, small bowel mesentery, spleen, lungs and mediastinum, albeit very rarely [[3], [4], [5], [6]]. When involving the gastrointestinal tract, it is most frequently located in the submucosal layer [5].

Patients with HP commonly present with non-specific complaints such as abdominal pain, change in bowel habits, loss of appetite/weight, and anaemia. HP exceeding 1.5 cm in size commonlypresent with symptoms of gastric outlet obstruction. HP can undergo complications that conventionally occur in normal pancreatic tissue such as acute and chronic pancreatitis, pancreatic abscess and pancreatic pseudocyst formation. Malignant transformation of HP is a rare occurrence reported in only 0.7–1.8% of HPs in the current literature [7]. There is a slight predominance for malignant transformation in women over men, with the mean tumor size being in excess of 3.5 cm and histology almost exclusively ductal adenocarcinomas [7]. Jaervi and Lauren proposed 3 criteria for the diagnosis of malignant transformation of HP: (1) The tumor having to be found within or close to ectopic pancreatic tissue (2) Direct transition observed between pancreatic structures and carcinoma with definitive exclusion of metastatic deposit or neoplastic invasion from adjacent organs (3) Non-neoplastic pancreatic tissue must comprise developed acini and ductal structures [8].

Differentials for HP on radiological investigations (Computer tomography (CT) and MRCP) include other submucosal neoplasms of the gastrointestinal tract such as leiomyomas or gastrointestinal stromal tumors. Several radiological characteristics of HP have, however, been reported by Kim et al. to be independently significant (p < 0.05) for diagnosis of HP: typical location (pre-pyloric antrum or duodenum), endoluminal growth pattern, ill-defined borders, prominent enhancement of overlying mucosa and an LD/SD ratio of >1.4. Their study goes further to state that when two of these 5 criteria are used in combination, the sensitivity and specificity for diagnosing HP is 100% and 82.5% [9].

On endoscopic ultrasound (EUS), features of ectopic pancreas include indistinct borders, lobulated margins, presence of anechoic duct-like structures, central umbilication, intramural growth pattern, and localization within two or more layers. Kim et al. reported that EUS features of HP that differentiate them significantly (p < 0.05) from mesenchymal tumors include: larger longest/shortest diameter ratio, antral location, mural growth pattern, third (submucosal) layer disruption, irregular margins, and intermediate echogenicity [8]. Previously, EUS-FNA was commonly superficial and non-diagnostic. However, with recent advancements in expertise, the sensitivity of EUS-FNA for diagnosis of HP has improved and been reported at 80–100% [10,11].

Histopathologically, the gross appearance of HP includes a characteristic central ductal orifice, pancreatic acini, ducts, islets of Langerhans, and intervening connective tissue.

The management of HP is controversial and commonly delayed due to diagnostic dilemma. Reasons for surgical treatment depend on the presence of symptoms, management of complications, excluding malignancy or simply diagnostic uncertainty.

## 4 Conclusion

Even though reports of patients with HP presenting with massive bleeding gastrointestinal tract and even malignancy can be easily found in the literature to date, HP is still most commonly an incidental finding. Ambiguity surrounding its diagnosis commonly gives rise to interventional dilemma and delay. While there have been many guidelines and reports detailing the characteristic features of HP on a myriad of imaging modalities, the gold standard for diagnosis remains that of EUS and FNA with histological confirmation. This case report has been written in concordance with the SCARE criteria [1].

## Conflicts of interest

Authors of this manuscript have no financial or personal relationships with other people or organizations that could inappropriately influence or bias our work.

## Sources of funding

There was no financial funding or sponsors involved in the collection, analysis and interpretation of data; in the writing of the manuscript; and in the decision to submit the manuscript for publication.

## Ethical approval

Ethical approval has been exempted by the institution in which this manuscript was written and submitted from. Written informed consent was obtained from the patient for publication of this case report and accompanying images. A copy of the written consent is available for review by the Editor-in-Chief of this journal on request. There is no ethical issue in this paper and all identifying names or identities have been omitted from the manuscript.

## Consent

Written informed consent was obtained from the patient for publication of this case report and accompanying images. A copy of the written consent is available for review by the Editor-in-Chief of this journal on request. There is no ethical issue in this paper and all identifying names or identities have been omitted from the manuscript. Ethical approval has been exempted by the institution as there are no patient identifiers or names included.

## Registration of research studies

This study does not involve human experimental subjects nor observational research

## Guarantor

The guarantor for this manuscript will be Dr. Brian Goh Kim Poh, the corresponding author.

## Provenance and peer review

Not commissioned, externally peer-reviewed.
